# Proteomic Changes of Alveolar Lining Fluid in Illnesses Associated with Exposure to Inhaled Non-Infectious Microbial Particles

**DOI:** 10.1371/journal.pone.0102624

**Published:** 2014-07-17

**Authors:** Laura Teirilä, Kirsi Karvala, Niina Ahonen, Henrik Riska, Anne Pietinalho, Päivi Tuominen, Päivi Piirilä, Anne Puustinen, Henrik Wolff

**Affiliations:** 1 Finnish Institute of Occupational Health, Helsinki, Finland; 2 Department of Pulmonology, Helsinki University Central Hospital, Helsinki, Finland; 3 Raasepori Health Care Center, Raasepori, Finland; 4 Unit of Clinical Physiology, Department of Clinical Physiology and Nuclear Medicine, Medical Imaging Center, Helsinki University Central Hospital, Helsinki, Finland; The Hospital for Sick Children and The University of Toronto, Canada

## Abstract

**Background:**

Hyperresponsiveness to inhaled non-infectious microbial particles (NIMPs) has been associated with illnesses in the airways. Hypersensitivity pneumonitis (HP) is considered to be the prototype for these NIMPs-related diseases; however, there is no consensus on the definitions or diagnostic criteria for HP and the spectrum of related illnesses.

**Methods and Findings:**

In order to identify the possible diagnostic markers for illnesses associated with NIMPs in alveolar lining fluid, we performed a proteomic analysis using a two-dimensional difference gel electrophoresis on bronchoalveolar lavage (BAL) fluid from patients with exposure to NIMPs in the context of damp building-related illness (DBRI) or conditions on the borderline to acute HP, designated here as agricultural type of microbial exposure (AME). Samples from patients with HP and sarcoidosis (SARC) were included for reference. Results were compared to results of healthy subjects (CTR). Western blot was used for validation of potential marker proteins from BAL fluid and plasma. Protein expression patterns suggest a close similarity between AME and HP, while DBRI was similar to CTR. However, in DBRI the levels of the inflammation associated molecules galectin-3 and alpha-1-antitrypsin were increased. A novel finding emerging from this study was the increases of semenogelin levels in BAL fluid from patients with AME, HP and SARC. Histone 4 levels were increased in AME, HP and SARC. Elevated plasma levels of histone 2B were detected in HP and SARC, suggesting it to be a potential blood indicator for inflammatory diseases of the lungs.

**Conclusions:**

In this study, the proteomic changes in bronchoalveolar lavage of DBRI patients were distinct from other NIMP exposure associated lung diseases, while changes in AME overlapped those observed for HP patient samples. Some of the proteins identified in this study, semenogelin and histone 4, could function as diagnostic markers for differential diagnosis between DBRI and HP-like conditions.

## Introduction

Certain occupations and surroundings increase the risk of exposure to inhaled non-infectious microbial particles, referred to as NIMPs in this article. NIMPs contain microbial cell wall structures and other microbial components, which are not infectious, but can activate immune response via Toll-like receptors and other pattern recognition receptors [1], [2]. Working in an agricultural environment, especially handling of moldy hay or grain, exposes farm workers to NIMPs. The exposure to the indoor air in damp buildings, will also cause exposure for NIMPs.

Hypersensitivity pneumonitis (HP), is a well-known yet complex syndrome associated with the exposure to NIMPs. HP is an inflammatory disease of the lung alveoli caused by exposure to a wide range of airborne substances: bacteria, fungi, even inorganic materials like certain chemicals. In some cases HP can also be caused by exposure to damp buildings [3]. Only a small portion of those exposed (1–15%), develop HP [4], [5]. According to a two hit hypothesis suggested to explain this: genetic factors (first hit) and exposure patterns (second hit) are required for the NIMP exposure to cause HP [6].

At least four separate sets of differing diagnostic criteria are currently in use for HP evaluation [7]. HP is traditionally divided in acute, subacute and chronic forms although there is no generally accepted definitions of these different and often overlapping forms of HP [6], [7], despite the long diagnostic history of this disease. Substantial diagnostic problems are also encountered with the severe fibrosing forms of HP which start to resemble other forms of interstitial pulmonary fibroses and with the less severe HP-like conditions which however do not fulfil all of the diagnostic criteria [8].

Damp building-related illness (DBRI) is a much less well-established illness than HP, even though a consensus exists that dampness and mould in buildings are a risk factor for health [9]–[11]. DBRI has been typically associated with prolonged exposure to water-damaged buildings, as in the case of the HP only some of those exposed will develop symptoms [12], [13]. The DBRI-associated symptoms tend to be diverse: fever, headache, myalgias, and respiratory symptoms like cough that in some respects resemble HP. After the initial phase an exposure to a damp building can result in persistent respiratory symptoms, especially asthma and increased sensitivity to quality problems in the indoor air (possibly due to sensitization to the organic or inorganic compounds associated with indoor air in damp buildings) even though the primary source of the exposure has been eliminated [14], [15]. There are no well-defined diagnostic criteria for DBRIs, and DBRI-specific diagnostic laboratory tests are lacking. Earlier studies of our group have shown that exposure to conditions of damp buildings increased lymphocyte levels in the BAL fluid of DBRI patients [16]. There is no specific quantity or quality of NIMPs which can be stated to designate an exposure state causing significant health effects. In other words, no specific causal agents of adverse health effects in damp buildings have yet been identified conclusively [11], [17].

Bronchoalveolar lavage (BAL) is widely used to obtain diagnostic information about interstitial lung diseases (ILDs) [18]. The BAL procedure provides unique insights into the condition of the alveolar spaces and thus BAL findings, like immune cell patterns, play an important role in supporting the diagnosis of interstitial lung diseases when combined with other clinical data and thoracic tomography [19].

Previous studies have reported that pulmonary disorders such as idiopathic interstitial fibrosis, sarcoidosis and asthma alter the protein composition of bronchoalveolar lavage [20], [21]. Analysis of BAL sample by two-dimensional gel electrophoresis has been shown to be a useful way of studying the pathogenesis of lung diseases [22]–[24].

This study originated from the clinical differential diagnostic problems posed by DBRI on the one hand and on the other agricultural NIMP exposures (AME) that often do not meet the criteria for HP. The study had two goals, the first was to examine the proteomic changes in alveolar lining fluid in conditions where exposure to NIMPs had resulted in a pathologic condition in order to discover possible diagnostic markers for this type of illness. The second goal was to examine the relationship between HP, AME and DBRI from a proteomic point of view.

We collected BAL fluid from patients with DBRI or AME with symptoms and findings that mostly fall short of classic HP. In addition, samples from HP patients were used as the reference material of an established lung disease associated with inhaled NIMPs, while samples from sarcoidosis (SARC) patients served as the reference material for a lung disease in which there is no direct association with NIMP exposure. Finally healthy non-smoking subjects served as controls. The two-dimensional difference gel electrophoresis (2D-DIGE) was used to identify the differences in protein expression between BAL fluids from different patient groups. Potential biomarker proteins were validated with Western blotting. The results revealed a clear difference in the protein expression patterns of alveolar lining fluid between DBRI and the other NIMP exposure associated groups: AME and HP. Four potential biomarkers (alpha-1-antitrypsin, galectin-3, histone H4, semenogelin I) were identified and these may be useful in monitoring the inflammatory state of the lung diseases after NIMP exposure, although the markers do not appear to be specific for illnesses associated with NIMPs.

## Materials and Methods

### Patients and collection of the BAL and plasma samples

BAL samples were from patients examined in the Finnish Institute of Occupational Health (FIOH) in 1997–2008. The patients had been sent to the occupational diseases clinic at the FIOH because of suspicion of occupation related respiratory diseases. The study targeted non-smoking patients with chronic symptoms (for more than one year) compatible with DBRI (n = 17) or symptoms related to agricultural exposure (AME, n = 9) for NIMPs. Those patients who had been treated with oral steroids during the past two months were excluded. An occupational physician experienced in indoor air problems conducted a retrospective evaluation of each patient’s exposure at work on the basis of available documents in the patient records. For the patients in the DBRI group, the documents included reports on building structure damage and, in most instances, microbial measurements of structural materials taken from the building. In order to be included in the study, the exposure to NIMPs had to be evaluated as being clear and significant. For the patients in the agricultural exposure group, AME, the criterion for NIMP exposure was handling of organic moldy material.

BAL fluids from patients with HP (n = 10) served as the reference material for an established lung disease associated to NIMPs, and sarcoidosis (n = 11), served as the reference material for a lung disease with no direct association with NIMPs, these specimens had been collected in an earlier study from the Meltola Hospital (a former pulmonary hospital) [25]. For HP the diagnostic criteria according to Terho [26] were used. In all of the HP cases acute symptoms were present and the illnesses can be considered to be in an acute phase [27]. The control group (CTR) of this study comprised of healthy individuals from the personnel of the Meltola Hospital (n = 16) and FIOH (n = 4) with no smoking histories. All tested plasma samples (available from CTR, HP and SARC groups) were from the Meltola Hospital. The remainder of the healthy controls had been obtained at the FIOH in year 2001 (n = 4/20). Both of these samplings of healthy controls were used in 2D-DIGE analysis. In the Western blot validation studies, six BAL samples from healthy volunteers from FIOH with positive smoking histories served as an additional control group for monitoring the effect of smoking.

In all cases the bronchoalveolar lavage was performed as described in [28]. After pooling of the BAL fluid, a 20 ml sample was separated and centrifuged at 1000 rpm for 5 minutes. The supernatant was frozen at −20°C. Sample freeze-thaw cycles were avoided by using sample aliquots. Both controls and disease samples were stored similarly. The investigated BAL samples were free of excessive blood- or epithelial contamination [25]. Patient characteristics and additional information about the BAL fluid content are given in Table 1. Major symptoms and high-resolution computed tomography (HRCT) findings of the DBRI and AME group patients are described in Table 2. Written consent for participation in this study was obtained for the samples taken from FIOH patients. The samples of patients from Meltola hospital were originally intended for and used in a different study and written or verbal consent was not required for this study. The Coordinating Ethics Committee at the Hospital District of Helsinki and Uusimaa reviewed and approved the study protocols including the use of the samples from the Meltola hospital (Diary number 320/13/03/00/09).

**Table 1:** Patient characteristics.

Diagnosis	DBRI^a^	AME^b^	HP^c^	SARC^d^	Controls	In total
**No. of cases (%)**	17 (25.4)	9 (13.4)	10 (14.9)	11 (16.4)	20 (29.9)	67
**Male# (%)**	3 (17.6)	3 (33.3)	8 (80)	4 (36.4)	9 (45)	27 (40.3)
**Female# (%)**	14 (82.4)	6 (66.7)	2 (20)	7 (63.6)	11 (55)	40 (59.7)
**Age**						
Median	48	47	54	48	35	45
Mean (SD)	48 (8)	48 (6)	53 (10)	45 (13)	36 (9)	45 (11)
Range	34–61	41–57	33–68	27–65	22–52	22–68
**Albumin mg/l BALfluid**						
Median	30	36	39	66	23	33
Mean (SD)	36 (22)	47 (29)	67* (63*)	82 (48)	23* (9*)	51 (24)
**Cells×10E6/l BALfluid**						
Median	122	278	280	114	125*	146
Mean (SD)	140 (73)	281 (127)	367 (340)	178 (111)	128 (61)*	200 (176)
**Lymphocytes %**						
Median	20	28	52	35	18*	22
Mean (SD)	25 (16)	31 (26)	52 (21)	26 (15)	18 (9)*	29 (20)
**Neutrophils %**						
Median	3*	2	2*	1*	1**	1
Mean (SD)	3 (3)*	6 (9)	3 (3)*	1 (1)*	2 (3)**	3 (5)

*Data available for ≥80% of samples.

**Data available for ≥75% of samples.

a Damp building-related illness.

b Agricultural type of microbial exposure.

c Hypersensitivity pneumonitis.

d Sarcoidosis.

**Table 2:** Major symptoms and HRCT findings.

	DBRI n = 17	AME n = 9
Cough	14	8
Dyspnea	13	8
Fever and chills	15	9
Duration of symptoms^a^		
0–5 years	9	6
6–10 years	4	1
>10 years	4	2
Asthma diagnosis	8	2
HRCT^b^ findings compatible with HP^c^/HRCT made	none/12	3/9

a before BAL procedure.

b HRCT = high-resolution computed tomography.

c Hypersensitivity pneumonitis.

### 2-DIGE and DeCyder analysis

Four-to six samples per study group were used in the 2D-DIGE. About 10 ml of each BAL sample was concentrated with ultrafiltration (Amicon Ultra-15 5000 MWCO, Millipore, Ireland) to 100 µl. The sample was then depleted of albumin and immunoglobulin G to facilitate higher sample loading, and to improve detection of low-abundance proteins. This procedure was done with the ProteoPrep Immunoaffinity Albumin and IgG Depletion Kit (Sigma Aldrich, St. Louis, MO, USA) according to the manufacturer’s protocol. After the determination of the protein concentration (BioRad kit) BALF proteins were precipitated with 2-D Clean-Up Kit (GE Healthcare) and dissolved to a protein concentration of 2 µg/µl in 7 M urea, 2 M thiourea, 4% CHAPS (Anatrace, Maumee, OH, USA) and 30 mM Tris-HCl, pH 8.8. Then, 10 µl from each sample was pooled for the internal standard and 40 µg of BALF proteins were labelled using 200 pmol Cy3 or Cy5 dyes (CyDye DIGE Fluor minimal dyes; GE Healthcare) according to the Ettan 2-D DIGE instructions. The Cy2 dye was used as an internal standard whereas Cy3 and Cy5 labellings were randomized evenly between study groups. For each combined Cy3- and Cy5-labelled sample pair, 40 µg of Cy2-labelled internal control sample was added prior to the quantitative 2D-DIGE analysis. CyDye labelled protein samples were separated by isoelectric focusing (IEF) using IPG strips (13 cm, pH 3–10 NL, GE Healthcare). The IEF strips were rehydrated 6 hours in 250 µL of the rehydration solution (7 M urea, 2 M thiourea, 4% CHAPS, 0.04% bromophenol blue (BPB) containing 0.5% IPG buffer, pH 3–10 NL (GE Healthcare) and 1.2% DeStreak reagent (GE Healthcare). Samples were absorbed onto the IPG strips by cup-loading. IEF was performed using Ettan IPGphor II (GE Healthcare) at 20°C using a limit of 75 µA/strip as follows: 3 hrs at 150 V, 2 hrs at 300 V, a linear gradient from 300 V to 1000 V for 6 hrs, the 2nd gradient from 1000 V to 8000 V for 2 hrs and finally 2 hrs at 8000 V (total 29 000 Vhrs). Strips were stored at −20°C and thawed at room temperature just before the SDS-PAGE analysis. They were incubated first for 15 min in a buffer containing 50 mM Tris–HCl (pH 6.8), 6 M urea, 2% SDS, 0,04% BPB and 30% glycerol with 1% dithiothreitol (DTT), followed by a 15 min equilibration in the same buffer with 2% iodoacetamide (IAA) instead of DTT. The two-dimensional SDS-PAGE was run on Criterion PreCast gels (10–20% gradient Tris–HCl gels, BioRad) in a Criterion Dodeca Cell (BioRad) with 200 V for approximately 75 minutes. Gels were scanned between low fluorescence glass plates using an Ettan DIGE Imager (GE Healthcare) at wavelengths of 480 nm for Cy2, 540 nm for Cy3 and 680 nm for Cy5 with 100 µm pixel size. After scanning, the gels were fixed in 30% ethanol, 1% acetic acid and silver stained [29]. The cropped images of 2-D DIGE gels were analyzed for differences in protein expression by means of internal Cy2 labelled standard using DeCyder 2D 7.0 software (GE Healthcare). Gel spots with at least a 1.5-fold spot volume ratio change and a Student’s *t*-test *p* value below 0.05 were picked for identification.

### Protein identification

Significantly up- or down-regulated protein spots were in-gel digested as previously described [30]. In brief, the protein spots were excised from the gels, reduced with dithiothreitol, and alkylated with iodoacetamide before in-gel digestion with trypsin (modified sequencing grade porcine trypsin, 0.04 µg/µl, Promega, Madison, WI, USA) for 16 h at +37 °C. After removing the supernatants to fresh tubes, the remaining peptides were extracted twice from the gel pieces by using 100 µl of 5% formic acid in 50% acetonitrile (ACN). The extracts were then dried in a vacuum centrifuge, and dissolved in 2% formic acid. Each peptide mixture was analyzed with an automated nanoflow capillary LC–MS/MS using CapLC system (Waters, Milford, MS, USA) coupled to an electronspray ionization quadrupole time-of-flight (Q-TOF) mass spectrometer (Waters). Reversed phase separations were accomplished with a 75 µm×15 cm NanoEase Atlantis dC_18_ column at a flow rate of 200 nl/min. Solvent A was 0.1% formic acid in 5% ACN, and solvent B 0.1% formic acid in 95% ACN. The peptide separation was achieved with a linear gradient of 0–60% of solvent B in 30 min.

The obtained mass fragment spectra were analyzed with in-house Mascot v.2.1 (Matrix Science Ltd., London, UK) and searched against human or mammalian entries in the NCBInr database. One missed cleavage was allowed, and searches were performed with fixed carbamidomethylation of cysteines, and variable oxidation of methionine, histidine, and tryptophan residues. A fragment tolerance of 0.2 Da and parent tolerance of 0.5 Da were used with trypsin as the specified digestion enzyme. A minimum number of two matched peptides or a Mascot score higher than 70, was considered significant.

### DeCyder Extended Data Analysis and GO classification

DeCyder Extended Data Analysis software (Version 7.0, GE Healthcare) was used for multivariate analysis of protein expression and protein Gene Ontology –classification based on the DeCyder 2D 7.0 software data of identified proteins. Protein spots not presented in at least 75% of the spot maps were removed from the analysis. Protein enrichment analysis was performed with a functional annotation tool: DAVID Bioinformatics Resources 6.7. (http://david.abcc.ncifcrf.gov/).

### Western blot

The relative protein expression of semenogelin I, histones 2B and 4, alpha-1-antitrypsin and galectin-3 in BALF (62 samples) and in plasma (37 samples) were evaluated by Western blot analysis to validate proteins identified from 2-DIGE and DeCyder analysis.

Volumes of 15 µl of BALF samples or 1.5 µl of plasma were run on 10–20% Tris-HCl Criterion gradient gels (Bio-Rad) at 100 V for 5 minutes, and 200 V for 60 minutes in running buffer (Biorad) of 25 mM Tris pH 8.3, 192 mM glycine and 0.1% SDS. A pool of all of the BALF or plasma samples was used as an internal standard on each gel to provide a reference from which to normalize the results. Two different molecular weight markers, Dual Color (Biorad) and MagicMark (Invitrogen, Carlsbad, CA, USA), were used in order to improve detection and analysis of protein bands. Proteins were transferred to polyvinylidene difluoride membranes (Millipore, Billerica, MA, USA) using the Criterion Blotter (Biorad) at 300 mA for 2 h in transfer buffer (25 mM Tris, 192 mM glycine, 20% methanol). Membranes were blocked with 5% skimmed milk in PBS, and incubated in a tube roller for 18 h at +4°C with polyclonal primary antibodies histone H4 (HIS4, anti-rabbit, dilution 1∶500) from Cell Signaling Technology, histone H2B (HIS2B, anti-rabbit, 1∶2000) and semenogelin I (SEM, anti-rabbit, 1∶1000) from Abcam, galectin-3 (GAL3, anti-rabbit, 1∶500) from Santa Cruz Biotechnology Inc and anti-α1-antitrypsin (a1AT, anti-mouse, 1∶4000) from AbFrontier. The Histone H2B antibody produced at best a weak signal in Western blots made from BAL samples. Only results obtained with the histone antibody H4 are shown for BAL samples. The H4 antibody did not produce any signal with the plasma samples (data not shown). Immunoblots were stained with polyclonal goat anti-rabbit or anti-mouse peroxidase-conjugated immunoglobulins (Dako Cytomaton, Glostrup, Denmark) and visualized with chemiluminescent HRP-substrate ECL detection reagent (Perkin Elmer, Waltham, MA, USA). The blots were imaged with ImageQuantLAS 4000 mini, software version 1.0 (GE Healthcare). Quantitation of the protein bands on immunoblots was accomplished by calculating the intensities of the bands with image analysis software v.7.0 ImageQuant TL from GE Healthcare.

Statistical analyses were performed using GraphPad Prism 5 software (GraphPad Software). A Mann-Whitney *U* test was used to compare the differences between the groups, since neither BAL nor plasma data is not normally distributed. A *p* value of <0.05 was considered to be statistically significant.

### ELISA measurements

IgG ELISA assay for the BAL samples was performed following the manufactureŕs instructions (eBioscience, San Diego, CA, USA). ELISA assay detected the total amounts of IgG.

## Results

### Proteomic analysis of bronchoalveolar lavage after exposure to inhaled NIMPs

**Figure 1:**
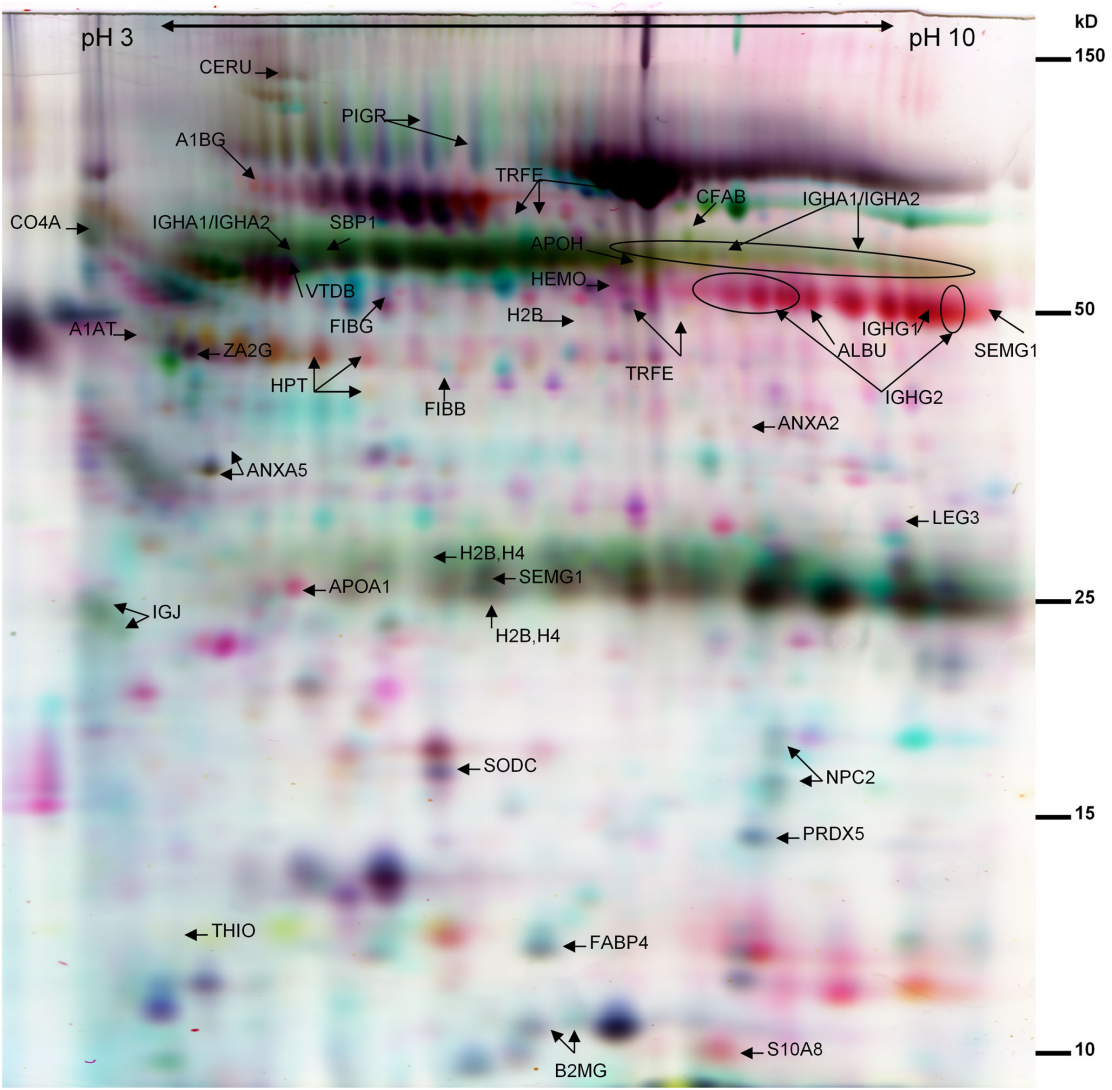
The false color image of two-dimensional DIGE gel of BAL. The gel image represents the Cy3 labeled (red), Cy5 labeled (blue) and Cy2 labeled (yellow) patient samples. The latter is a pooled sample, which served as an internal standard. Spot abbreviations refer to the identified proteins listed in Table S1. Molecular weights are shown on the right edge of the gel and the pI range on the top part of the SDS-PAGE.

**Figure 2:**
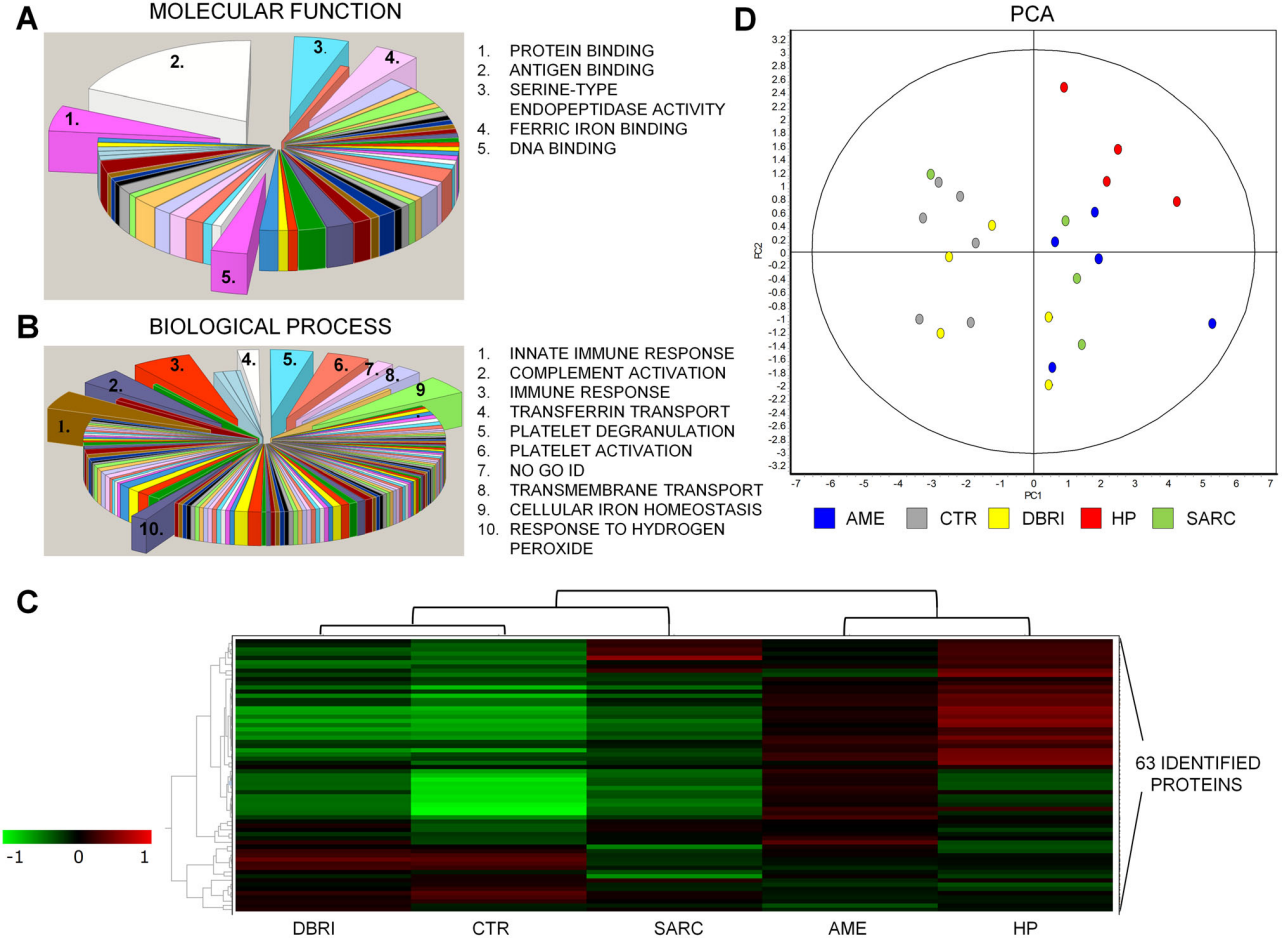
Protein classification and study group clustering. Classification of the proteins for the different gene ontologies: (A) molecular functions and (B) biological processess. The groups including ≥4 proteins from the set are depicted in the picture. (C) The hierarchical clustering of the study groups is displayed in the form of a heatmap. A dendrogram at the top of the heatmap represents a hierarchy of studied groups based on the degree of similarity in their protein expression. The heatmap shows the mean of a proteińs expression on all analysed spot maps in that particular experimental group. The protein expression levels of 63 identified proteins in the study groups are compared to levels of internal standard, red colour indicating upregulation and green colour indicating underregulation. Data clustering is based on Pearson distance (Pearson correlation, average linkage). (D) The clustering of the spot maps according to the variance of their protein expression was performed with principal components analysis. The first principal component (PC1) explained 59.8% of the variance and the second (PC2) 12.8%. Cumulative variance 90% was reached after 7 components.

Screening of potential biomarkers was performed for a total of 24 BAL samples from the healthy control (CTR n = 6) and four disease groups (AME n = 5, DBRI n = 5, HP n = 4, and SARC n = 4). DeCyder software detected on average 2000 spots per DIGE gel. A total of 63 proteins spots and 34 different proteins were identified from the selected gel spots (Figure 1 and Table S1), which were differentially expressed (Students t –test <0.05, a fold change ≤−1.5 or ≥1.5) between CTR versus one or all of the study groups (AME, DBRI, HP, SARC). Some of the differences were obtained also from the comparisons between DBRI versus AME. According to the Gene Ontology classification, most of the proteins were grouped as belonging to the extracellular region. One third of the proteins were associated with antigen binding. Identified proteins were also associated with protein binding, serine-type endopeptidase activity, ferric iron binding and DNA binding (Figure 2A). The outcome of the classification of proteins in biological processes pointed to the following categories: immune response, function of platelets, cellular iron ion homeostasis, transmembrane transport and response to reactive oxygen species (Figure 2B). Based on the protein enrichment analysis, extracellular secreted proteins, especially plasma proteins, were abundant among the identified proteins from the BAL samples (Table S2). Most of the proteins were also classified as glycoproteins, a common property of human biofluid proteins, and they contained a signal peptide for secretion.

### Differences in protein expression pattern of damp building-related illness in comparison to the other NIMP exposed groups

A hierarchical clustering was performed for the 63 identified proteins to compare the protein expression patterns between the experimental groups (Figure 2C). The CTR, DBRI and SARC study groups formed one main cluster with the other main cluster being comprised of AME and HP. Control and DBRI formed their own subgroup which excluded SARC, i.e. evidence that there was a similar overall protein expression patterns between these two groups. Marked upregulation of proteins was observed in the AME and HP experimental groups, and this feature distinguished these two groups from the other main cluster. The clustering of the spot maps according to the variance of their protein expression was performed with principal components analysis (Figure 2D). The homogeneity or heterogeneity of the protein abundance within and between the study groups is visualized in the format of a heatmap in Figure S1. The lymphocyte percentage of BAL samples chosen for DIGE analysis from DBRI group differed from the control samples (Student’s t -test <0.05), but not from the other disease group samples (Figure S2).

The proteins, which have 1.5 times lower or higher expression in study groups compared to control group are represented in number sets by the Venn diagram (Figure S3). Information about the identified proteins is shown in table S1.

Haptoglobin (HPT, this abbreviation refers to the abbreviation used in Table S1), histone variants, immunoglobulin G (IGHG2, IGHG1) and semenogelin (SEMG1) were overexpressed in all disease groups. None of the identified proteins were less abundant in DBRI as compared to CTR group. Two proteins, a component of the class I major histocompatibility complex, beta-2-microglobulin (B2MG) and an intracellular cholesterol transporter, epididymal secretory protein E1 (NPC2) were downregulated in AME, HP and SARC compared to control group.

Peroxiredoxin 5 (PRDX5) appeared to be underexpressed in SARC patients. Apolipoprotein A1 (APOA1) was upregulated in all microbial-associated lung conditions, but not in SARC.

None of the proteins in DBRI group seemed to be characteristic to that particular condition. The expression of protein S100-A8, which is a part of calprotectin, an acute phase reactant in inflammation, was elevated only in AME as compared to healthy persons. The amounts of complement factor B (CFAB) selenium binding protein (SBP1) and hemopexin (HEMO) were markedly increased, whereas those of superoxide dismutase (SODC) and thioredoxin (THIO) were reduced in HP compared to control. However, the levels of these proteins in AME and HP did not differ significantly from most of the other disease groups (data not shown).

Pulmonary surfactant proteins have been linked to innate immunity of the lung helping to prevent the infection and inflammation. However, no statistically significant differences in the expression of the identified surfactant proteins (SP-A and D) were detected between studied samples.

### Validation of potential indicators for NIMP associated lung diseases

**Figure 3:**
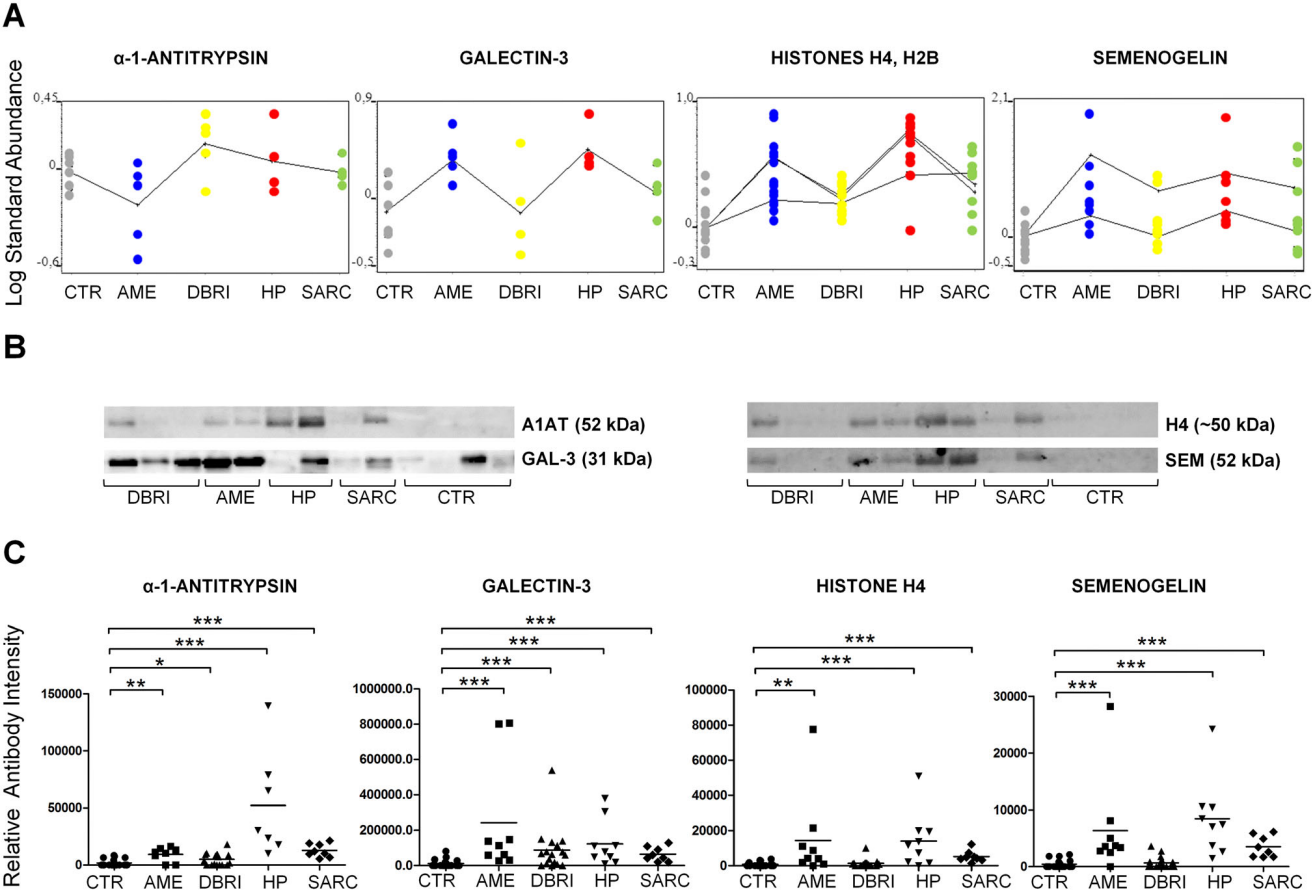
Immunoblot validation of protein markers in BAL. (A) Differential expression analysis based on DeCyder data for chosen protein markers. Every dot indicates the identified protein spot and its expression level in that particular study group. The difference in protein expression (average expression) between the groups is visualized with a continuous curve. Histone and semenogelin plots contains more than one curve due to several protein spot identification for histones H4, H2B (n = 3) and semenogelin I (n = 2) as shown in Table S1. (B) Images of immunoblot membranes and (C) immunoblot based validation results for protein markers. The number of samples in validation: CTR n = 19, AME n = 7–9, DBRI n = 17, HP n = 9, SARC n = 8. The means (black lines) are shown in scatter plots. *Indicates statistical significance, which is shown between experimental and control group, at the level of p≤0.05, **p≤0.01 and, ***p≤0.001.

The original reason for the study was to identify markers for diseases associated with the exposure to NIMPs, however, we did not find this type of marker (i.e. those displaying consistent increases over control levels in the DBRI, AME, and HP but not the SARC group; preferably so that the marker would not be implicated in other inflammatory conditions). In the validation study we concentrated on those markers that increased with reasonable robustness compared to the healthy controls. An additional restriction for western blot validation was provided by the availability and functionality of antibodies. Four proteins were chosen for immunological validation and quantitation: alpha-1-antitrypsin, galectin-3, histone H4 and semenogelin 1 (Figure 3).

Alpha-1-antitrypsin (A1AT) is a protease inhibitor; its levels may increase in the lungs during the inflammation and dysfunction of this protein has been claimed to be associated with several respiratory conditions. All the patient groups had significantly elevated A1AT levels as compared to healthy controls according to immunoblot quantitation results. The highest amounts of A1AT were detected in samples from HP patients (Figure 3C).

Galectin-3 has important roles in the innate immunity response such as the activation and chemoattraction of inflammatory cells. The levels of this protein were upregulated in HP and AME groups based on DeCyder analysis (Figure 3A, Table S1), indicating that it is one of the proteins acting similarly in these two hierarchical linked groups. Immunoblot revealed that there were significant changes between the experimental groups and healthy controls. The highest levels of galectin-3 were seen in AME patients (Figure 3C). There were no significant changes between the experimental groups (AME, DBRI, HP, SARC), when they were compared to each other.

Semenogelins are proteins which are known to participate in the formation of sperm coagulum. However, semenogelins have also been reported to be present in the lungs and in small cell lung carcinoma [31]. Based on DeCyder analysis, the level of semenogelin was upregulated in all of the studied disease patient groups (Figure 3A, Table S1). The immunoblot validation verified that the levels of semenogelin were markedly increased in AME, HP, and sarcoidosis patients as compared healthy controls. In the DBRI group, there was no significant difference in semenogelin abundance as compared to healthy controls (Figure 3C). The difference between DBRI and AME/HP was significant (p = 0.0009 for DBRI vs. AME and p = 0.0001 for DBRI vs. HP).

Histones are core components of nucleosomes and can boost inflammatory response by functioning as endogenous danger signals in our immune system [32]. Histone variants H2B and H4 were upregulated in all previously mentioned experimental groups, albeit less in DBRI according to differential expression analysis (Figure 3A, Table S1). The detected histones in BAL samples were aberrant in size. The spots of 20–25 kDa and 40–45 kDa size were identified as histones H4 and H2B in 2DE-DIGE gel analysis in the human BALF samples. The immunoblot based quantitation analysis of histone H4 in BAL fluid was performed on a protein band of approximately 50 kD (H2B was not measurable, see materials and methods). This aberrant size histone detection with the histone primary antibody used was confirmed by mass spectrometric analysis for this particular band from the immunoblot membrane. The increased amount of histone H4 in BAL fluid was associated with AME, HP and SARC (Figure 3C). The difference between DBRI and AME/HP was statistically significant (p = 0.01 for DBRI vs. AME and p = 0.0031 for DBRI vs. HP).

All the patients in the studied groups were nonsmokers. We also investigated the effect on smoking on the proteins selected for validation. Western blotting based validation was performed for six BAL samples obtained from healthy controls with smoking backgrounds. Only the levels of galectin-3 were elevated in a group of smokers as compared to controls (CTR), p = 0.0482 (data not shown).

To study the immunoglobulin response in lungs of patients exposed to microbial particles the concentration of total IgG was measured with ELISA assay from native BAL. The elevated immunoglobulin levels were observed in BAL fluids from the AME, HP and SARC groups (Figure S4).

### Elevated histone levels in plasma samples of hypersensitivity pneumonitis and sarcoidosis

**Figure 4:**
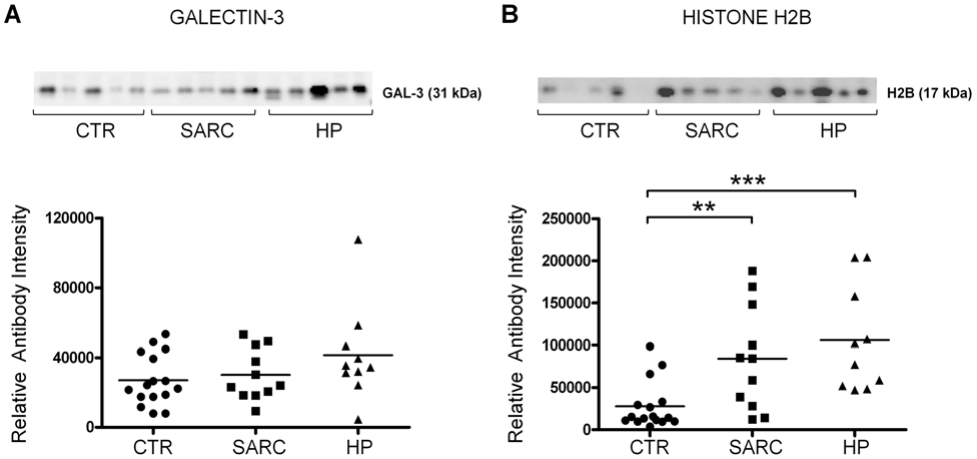
Immunoblot validation of protein markers in plasma. The immunoblot and validation analysis for (A) galectin-3 and (B**)** histone H2B from plasma samples. The number of samples in validation: CTR n = 16, HP n = 10, SARC n = 11. The means are shown in scatter plots. *Indicates statistical significance, which is shown between experimental and control group, at the level of p≤0.05, **p≤0.01 and, ***p≤0.001.

Histone H2B and galectin-3 levels were also analyzed from plasma samples of patients from control, HP and SARC groups. Validation of other chosen protein markers produced inconsistent results (data not shown), possibly due to interference of the similar size serum albumin or to low levels of target protein. There were no significant differences between the groups in galectin-3 levels, however, H2B levels were markedly higher in plasma of HP and SARC (Figure 4). Between HP and SARC there were no significant differences in the abundance of H2B. In plasma, the size of the histone H2B detected was 17 kDa, the predicted size of histone when it is a part of the nucleosome.

## Discussion

The results from the 2D gel analysis and the immunoblot validation studies point to a substantial difference in protein expression between the DBRI group and the AME/HP group. Similar protein expression patterns of AME and HP samples suggest there is a close association between AME and HP, while the protein expression pattern of DBRI resembled more that of healthy individuals and the SARC group.

Comparing then NIMP associated illnesses in order to understand the differences in proteomic outcome, they share a similar general pattern of symptom free periods followed by exacerbations when exposed to the appropriate NIMPs. In this study the time from the start of symptoms to the BAL procedure for DBRI and AME were comparable. The main difference is that in DBRI, the exposures are known to be substantially less intense than those experienced by subjects in the HP or AME groups and this could be a part of the explanation. The major symptoms for both DBRI and AME were fairly similar in this study, but there is a difference in that asthma is more often present in DBRI. In the literature HP is associated with a limited set of symptoms (fever, cough, dyspnoea) while the symptoms of DBRI are very variable and include symptoms from the upper airways, the nasal area, eyes and skin. A relatively obvious explanation for the differences between DBRI and HP is that they could affect separate parts of the airways, HP is known to affect the alveolar spaces while DBRI seems to affect the more proximal airways.

This study intended to resolve the diagnostic problems associated with DBRI and AME in our clinic. In order to provide reference material from HP and SARC, which are rarely encountered at the FIOH clinic, we used BAL samples collected from an earlier study [25]. HP is becoming a rarity in Finland and we consider ourselves fortunate to have access to these samples. The samples have been collected using an identical protocol to that used in the FIOH. All the samples have been stored and handled similarly and they have not been thawed and re-frozen, which has been shown to affect the stability of the proteins most [33]. There are substantial differences in the storage times between the samples from FIOH and those from the Meltola Hospital. However, no significant difference between the levels of validated proteins was observed between the healthy control samples from FIOH and those from Meltola Hospital (data not shown). It should also be noted that the protein expression pattern in the HP group mirrored that of the closely related AME group. If protein degradation were to be an important factor then one would not expect the protein levels in the HP and SARC groups to be greater than those in the DBRI group. Our protein identifications also seem to be comparable with the 2-DE results reported earlier from BAL collected from patients with interstitial lung disease [23]. We are thus confident that differences in the storage times did not influence the results in any substantial manner.

In general, there was less extensive lymphocytosis in the DBRI samples than in the case of AME and HP (Table 1). The total cell numbers and proportion of lymphocytes exceeded the normal range only in half of our studied DBRI research samples. However, samples chosen for DIGE analysis from DBRI group did display lymphocytosis comparable to the AME and HP groups (Figure S2) indicating that additional factors other than the numbers of inflammatory cells were responsible for the difference between the DBRI and AME/HP groups. Thus the proteomic analysis suggests that the DBRI, at least its usual clinical presentation, is distinct from HP and HP-like conditions. However, it is possible that the proteomic pattern in an initial phase of DBRI display more similarities with HP and AME, in addition the exposure to damp buildings can sometimes cause clinical HP [16].

We have earlier shown that DBRI is associated with a lymphocytosis in BAL fluid [16]. In this study the relative lack of protein changes in DBRI, although lymphocytosis was present, can be seen to be in parallel with an earlier report where no HP ensued in asymptomatic dairy farmers although they demonstrated increased BAL lymphocyte levels because of exposure to moldy hay [34]. Thus the lymphocytosis in DBRI can be seen as a non-pathologic response to inhaled NIMPs, not associated to an illness like HP.

It would definitely be interesting to compare the proteomic changes of a NIMP exposure in those persons that did develop an illness with those that did not. There are however a number of practical and ethical considerations which would make this an extremely difficult proposition. In fact the study mentioned earlier [34] is the only one where BAL has been obtained from this type of healthy NIMP exposed individuals. Thus our study is limited to pathologic conditions associated with the exposure to NIMPs, not the normal response to inhaled NIMPs.

Samples from patients with sarcoidosis served as a reference for a lung disease which has no direct association with NIMP exposure. However, an epidemiologic relationship has been observed between rural living and sarcoidosis [35]. According to DeCyder data the protein expression pattern of SARC resembled more that of control and DBRI than AME and HP, whereas the levels of immunoblot validated proteins showed similar kinds of elevation in both SARC and HP groups. This apparent discrepancy could be explained by the fact that the DeCyder process does not necessarily identify all proteoforms, which can be detected by antibody-based methods.

When collecting a BAL specimen from a patient with interstitial lung disease active lung infections are avoided as they will make the interpretation of the results almost impossible. Accordingly, there was no significant neutrophilia associated with any of the groups (Table 1). This more or less excludes the possibility that the measured changes were influenced by infections. Albumin levels did vary between the groups and was highest in the SARC group and the lowest in the CTR group (Table 1). It is well known that albumin levels increase in interstitial lung inflammations and can be seen to validate the selection of the patient groups [36], [37].

Immunoglobulin G levels are often used in clinical practice to help in the evaluation of the BAL samples. In our study, the IgG expression levels were high in the patients exposed NIMPs in agricultural environment and in patients suffering from sarcoidosis or hypersensitivity pneumonia as compared to healthy persons. These results are consistent with the earlier results reported in the literature [38] and indicate that the BAL fluid samples used were truly representative.

In the validation study, we selected the markers that increased with reasonable robustness relative to the healthy controls. The selected proteins included one novelty and other proteins “without” novelty i.e. molecules that could be considered as being established inflammatory markers. The proteins were validated from BAL samples of all five study groups, and from the plasma samples available from CTR, HP and SARC groups.

The most interesting finding was the detection of semenogelin I in BAL fluid in AME and HP, and also in sarcoidosis patients. Attempts to validate the semenogelin from plasma gave inconsistent results, possibly due to too low levels of the target protein. Semenogelin I and II are the major products secreted from the glandular epithelium of the seminal vesicles and the epididymis, that together with fibronectin, give rise to the gel-like coagulum of the newly ejaculated semen [39]. In addition, fragments of semenogelin have been claimed to possess antimicrobial effects [40], [41] and heparin binding properties in human seminal plasma [42]. It has also been observed that semenogelin has a high zinc ionbinding capacity thus possibly functioning as a regulator for zinc homeostasis [43]. Semenogelin expression has also been observed in non-genital tissues including trachea and bronchi [44]. Semenogelin I and II were identified in asthmatic chronic rhinosinusitis nasal lavage fluid, but were not observed in allergic rhinitis nasal lavage fluid [45]. There are also studies pointing to the involvement of semenogelins in different type of cancers such as small cell lung carcinoma [31], [46], [47]. Our observation seems to be the first demonstration where semenogelin is identified in bronchoalveolar lavage fluid of interstitial lung disease patients and patients exposed to inhaled non-infectious microbial particles. One possible role for semenogelin is its regulation of mucus viscosity which could be seen as a component of microbial defence. For example, changes in mucus viscosity may impair mucus clearance in the airways and lungs. Deficient mucus clearance is associated to lung bacterial infections and other respiratory diseases [48]. Regulation of zinc homeostasis is another possible mechanism of action. The lungs are directly exposed to higher oxygen concentrations than most other tissues and it has been shown that depletion of labile zinc renders airway epithelial cells highly susceptible to the apoptosis induced by oxidants [49], [50]. The allergic inflammation is also linked to perturbation in zinc homeostasis [51].

For histone component H4, significantly increased expression in BAL fluid was associated with AME, HP and SARC patient samples. Interestingly, the levels of other histone variant, H2B were increased in plasma samples of HP and SARC patients, whereas H4 we could not detect from plasma. Histones form the core component of the nucleosome, which is the central part of double-represented histones H2A–H2B and H3–H4 [52]. The small amounts of nucleosomes found in serum of healthy persons are attributable to release upon physiological cell death. Elevated levels of nucleosomes are observed in blood in diseases in which enhanced cell death has been reported to be present [53]. Elevated levels of histones have been observed in association with a common lung disease, chronic obstructive pulmonary disease (COPD) [54]. Histones function as damage associated molecular patterns (DAMPs) which trigger inflammation and they have been shown to play a central role in hyperinflammatory syndromes, mediating death in a mouse model of sepsis [32], [55]. Based on the present results of elevated histone levels in BAL and plasma it can be speculated that histones may play a role in the pathogenesis of interstitial lung diseases or conditions associated with inhaled NIMPs.

The size of detected histones in BAL samples were aberrant, which might be due to altered post-translational modification of these proteins. Post-translational modification of histones by phosphorylation, acetylation, methylation and ubiquination are processes which regulate chromatin structure and gene expression. Histone 4 has also been shown to be modified by SUMO (Small Ubiquitin-like Modifier), which can modulate deacetylase activity and thus influence transcriptional repression [56]. The attached SUMO proteins can increase the molecular weight of the other proteins depending on their number in the linked chain [57]. It needs to be clarified whether inhaled NIMPs can cause the appearance of aberrant size histones in BAL fluid and possible activation of post-translational modification.

The levels of galectin-3 were elevated in all of the studied lung diseases, with the highest expression of galectin-3 being observed in AME patients. Interestingly in DBRI, the galectin-3 levels were increased with a higher degree of statistical significance than for alpha-1-antitrypsin, which is a well-known indicator of lung inflammation. In contrast, there were no significant differences in galectin-3 levels compared to healthy controls in plasma samples of HP and SARC patients. Galectins are highly conserved family of lectins, which have binding affinity for β-galactosides [58]. During the tissue damage cytosolic galectins are passively released by dying cells or actively secreted by inflammatory activated cells [59]. Galectin-3 is considered as a potential damage associated molecular pattern (DAMP) and can also act as pattern recognition receptors (PRR), which binds to the glycans present on the cell walls of microorganisms [59]. Galectin-3 is involved in many innate immune response activating processes: chemoattraction of monocytes and macrophages [60], oxidative burst of neutrophils [61] and mast-cell degranulation [62]. Earlier studies have been shown increased levels of galectin-3 linked to inflammation or microbial infection in lungs. It was also reported to play an essential role in the development of lower airway hyperresponsiveness [63]–[66]. In acute inflammation, galectin-3 is increased and seems to have a pro-inflammatory role. However, if the inflammation progresses to a more chronic stage, galectin-3 has been reported to facilitate the walling-off process of tissue injury with fibrogenesis and organ scarring [67]. There are reports where galectin-3 has been utilized as a marker to follow the inflammatory activity of a disease or used as a factor for prognosis in cancers or chronic heart failure [68]–[70]. Serum levels of galectin-3 have been used to monitor the inflammatory stage of inflammatory bowel disease [71]. Our finding that the expression of galectin-3 is increased in HP and SARC in BAL is in line with its role in inflammation.

The BAL levels of alpha-1-antitrypsin (A1AT) were elevated in all groups with illnesses including DBRI. We could not detect A1AT from plasma possibly due to too low levels of the target protein. A1AT is an inhibitor of serine proteases and it is one of the plasma proteins which make up the acute phase proteins. One important physiological function of antitrypsin is to protect the lower respiratory tract against proteolytic destruction by human leukocyte elastase (HLE) [72]. A1AT deficiency may cause severe emphysema [73]. Patients with A1AT deficiency are also known to be hyperresponsive to organic dust exposure [74]. Recently, A1AT has been shown to have anti-inflammatory and tissue protective properties independent of its protease inhibitory effects [75], [76]. A1AT appears to be antibacterial and to act as an inhibitor agent in viral infections [77], [78]. It is mainly synthesized in liver, but interstitial A1AT in lungs is primarily produced by lung type II alveolar epithelial cells [79]. A1AT has been detected and found to increase in BAL of patients with interstitial lung disease in previously published proteomic studies of lung diseases [80]–[82] and its levels increase in association with different pulmonary exposures [83].

The healthy lung is classically thought to be sterile but recent studies have suggested that the lung possesses its own microbiome [84], [85], thus lung is in continuous interaction with microbial structures of its own microbiome. It has been demonstrated that there are differences in microbial composition of the airway flora between healthy lungs and those with chronic lung diseases, such as asthma, chronic obstructive pulmonary disease as well as cystic fibrosis [85]–[87]. In the future, it would be interesting to study the possible alterations in the lung microbiome associated with exposure to NIMPs and its contribution in protection or progression of this type of lung diseases.

## Conclusions

Taken together, the results from the 2D-gel analysis and the validation studies suggest that at least the typical case of DBRI, where the main symptom is asthma and/or an increased sensitivity to poor indoor air quality, is not closely related to AME and HP. This result was somewhat unexpected as all of these conditions are associated with the exposure to NIMPs and that the symptoms between the DBRI group and the AME group are rather similar. A possible explanation for the difference of DBRI and AME is that they affect separate parts of the airways, HP is known to affect the alveolar space while DBRI seems to affect the more proximal airways. We could detect increases in the levels of several proteins of interest that could be considered as markers of inflammation. Increases in the levels of some indicators of inflammation (alpha-1-antitrypsin, galectin-3) could also be demonstrated in the DBRI group. However, none of the identified proteins can be considered as being specific for illnesses associated with NIMPs. One of the novel findings was the high levels of semenogelin detected in BAL fluid from patients suffering from HP or AME. The detectability of histones (H2B) in plasma in HP and SARC suggest H2B to be a marker for the inflammation in the lungs. In future, prospective studies it would be interesting to analyze histones in blood also in DBRI and AME groups. As histones have been claimed to possess a pathogenic role in severe inflammations (sepsis) development of histone specific therapies and monitoring may be beneficial. The study also demonstrates the advantages of using of bronchoalveolar lavage samples for proteomic studies of changes in alveolar lining fluid associated to exposure of non-infectious microbial particles. Viewed from a clinical point of view, these results suggest that the BAL analysis of protein markers (including semenogelin I, H4 and total IgG) could be helpful in the differential diagnosis between DBRI and HP, especially when lymphocytosis is present in the BAL fluid. Furthermore, the determination of galectin-3 levels in BAL fluid might be useful for evaluating the condition of DBRI patients.

## Supporting Information

Figure S1 **Hierarchical clustering of spot maps.**
(TIF)

Figure S2 **Lymphocyte percentage in BAL samples chosen for DIGE analysis.**
(TIF)

Figure S3 **Expression of identified proteins.**
(TIF)

Figure S4 **Immunoglobulin G concentrations of BAL.**
(TIF)

Table S1 **Identified proteins.**
(XLSX)

Table S2 **DAVID enrichment analysis.**
(XLSX)
