# Supplementary figures and images for: Proteomic Changes of Alveolar Lining Fluid in Illnesses Associated with Exposure to Inhaled Non-Infectious Microbial Particles
Source: PLoS One. 2014 Jul 17;9(7):e102624. doi: 10.1371/journal.pone.0102624 (PMC4102538; doi:10.1371/journal.pone.0102624)

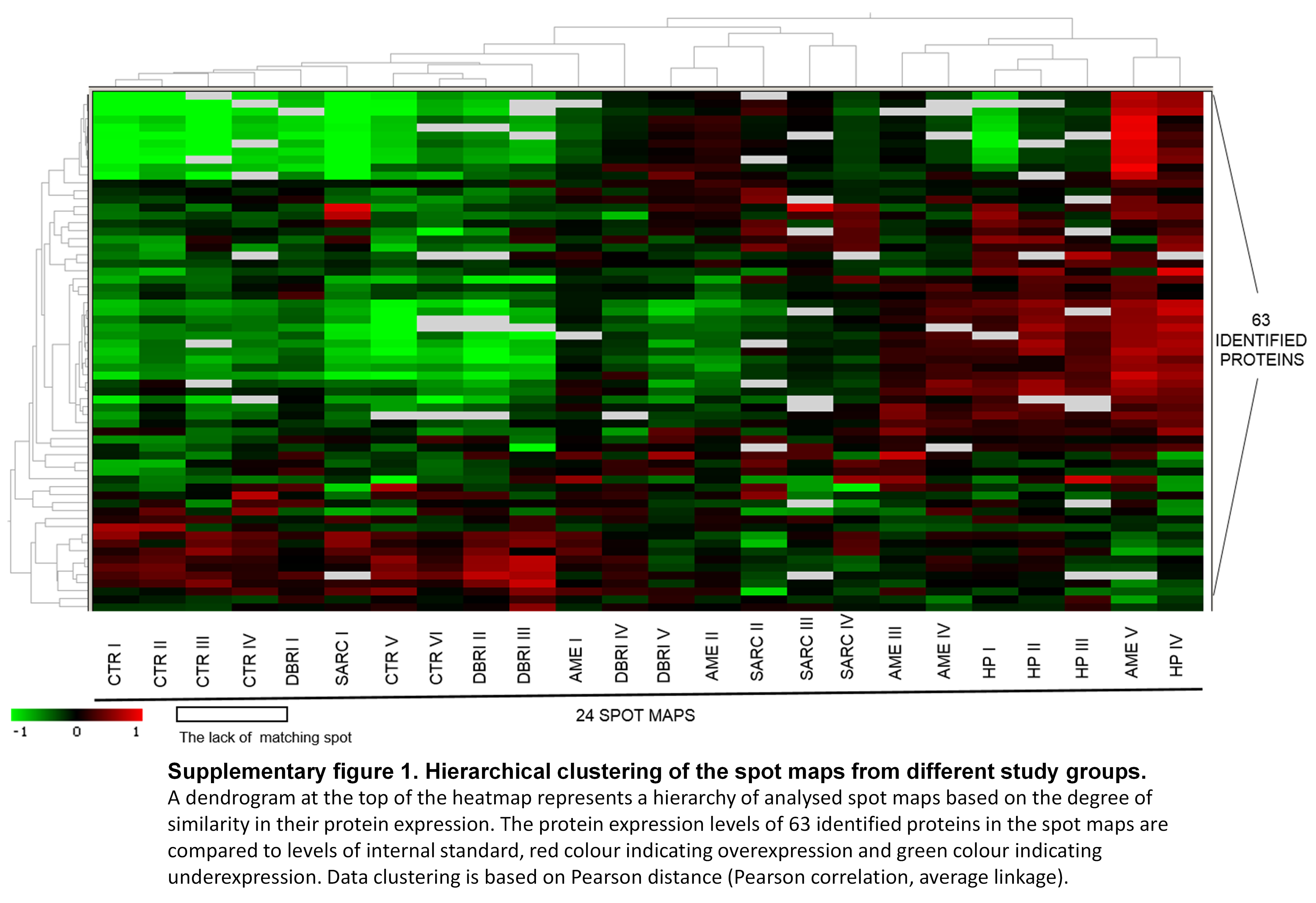

Supplement: Figure S1 — Hierarchical clustering of spot maps. (TIF) [file pone.0102624.s001.tif]

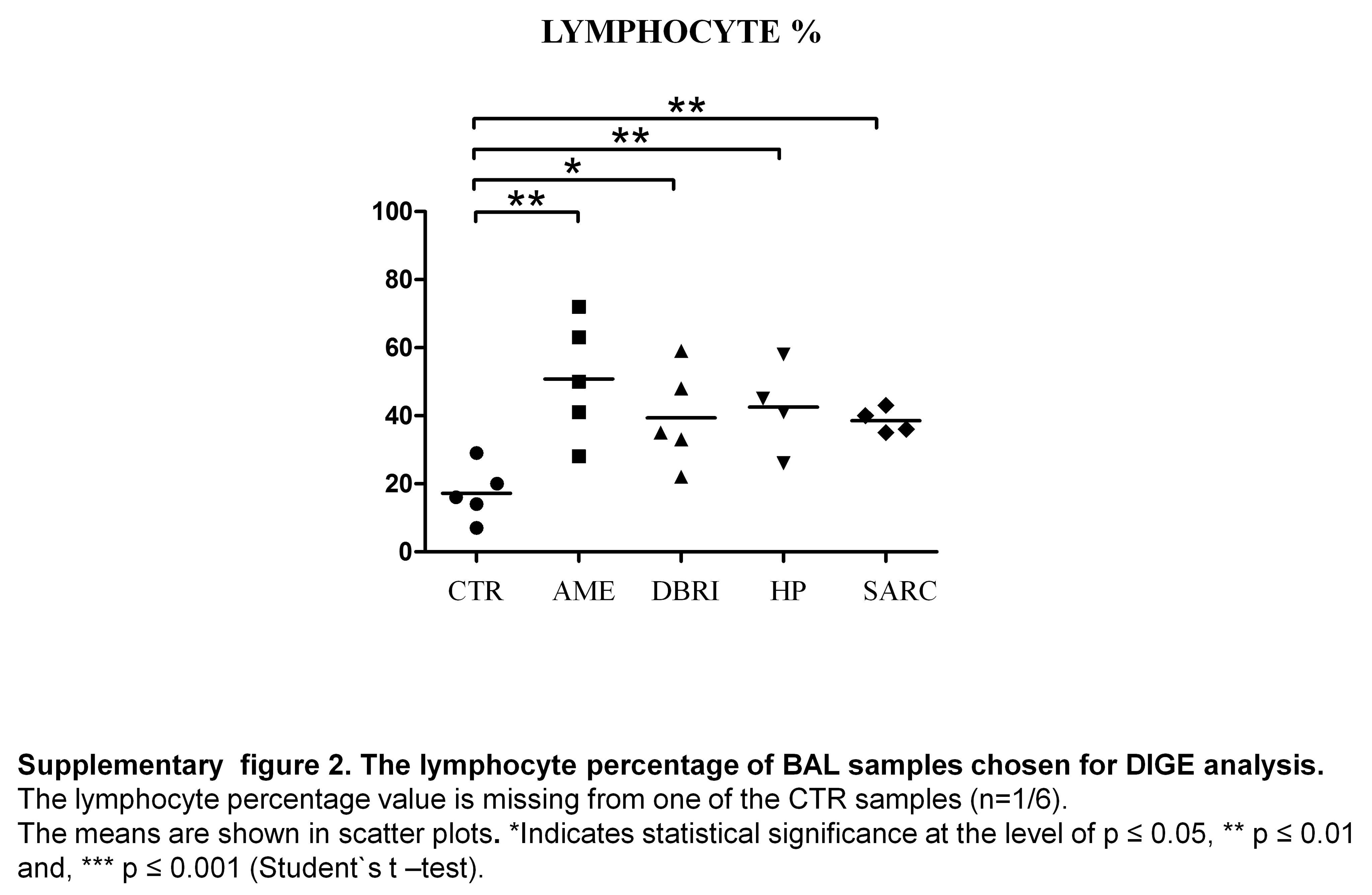

Supplement: Figure S2 — Lymphocyte percentage in BAL samples chosen for DIGE analysis. (TIF) [file pone.0102624.s002.tif]

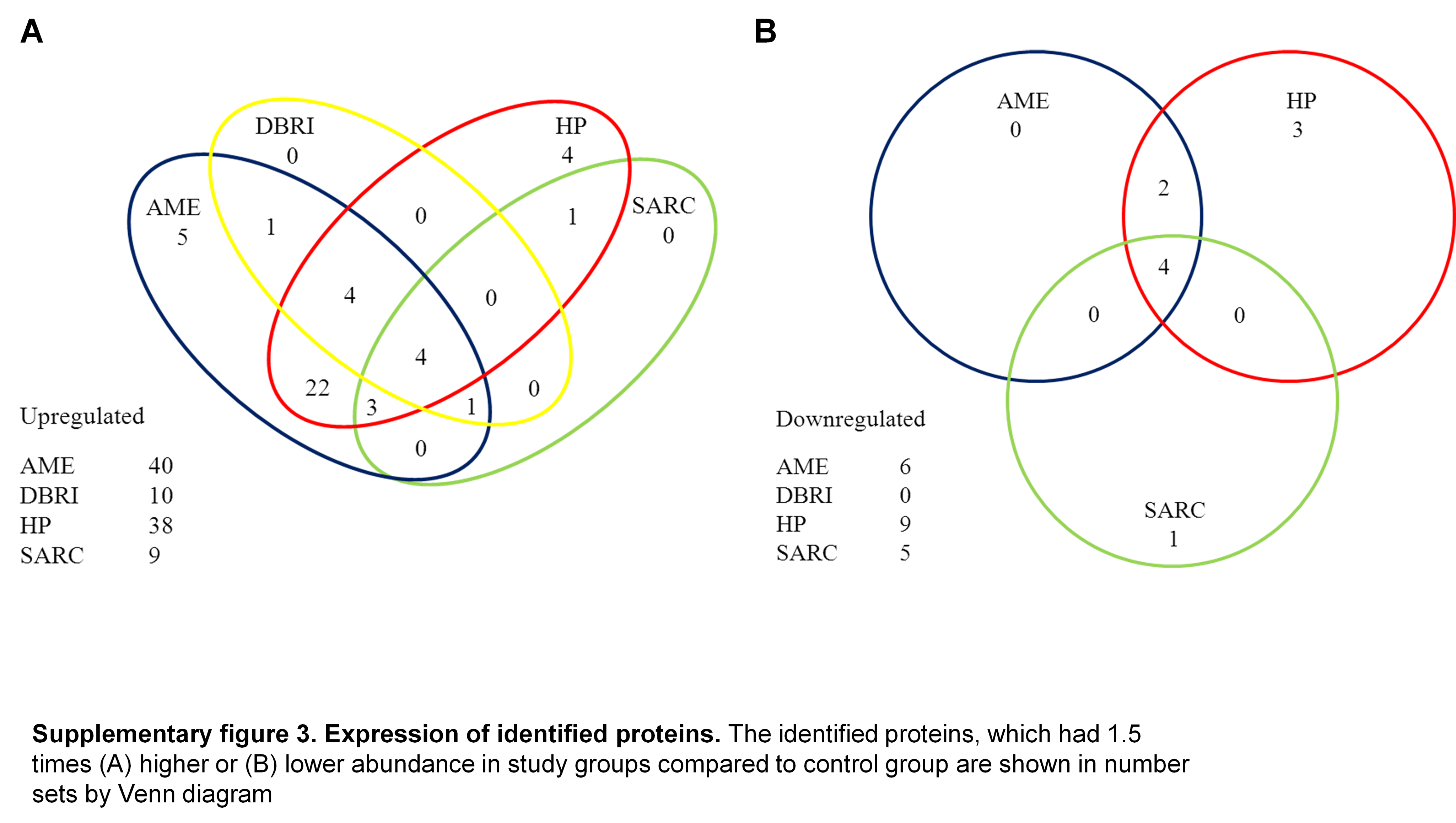

Supplement: Figure S3 — Expression of identified proteins. (TIF) [file pone.0102624.s003.tif]
